# Investigation of the Effect of Fe_3_O_4_ Grain Size on the Catalytic Degradation Performance of PAL/Fe_3_O_4_ Composites Using the W.-H. Method

**DOI:** 10.3390/ma19112226

**Published:** 2026-05-25

**Authors:** Hao Huang, Fei Xie

**Affiliations:** College of Mining, Guizhou University, Guiyang 550025, China

**Keywords:** catalytic activity, PAL/Fe_3_O_4_ composite, grain size, Williamson–Hall method

## Abstract

This study systematically investigated the preparation conditions of palygorskite/Fe_3_O_4_ composites. The grain size of Fe_3_O_4_ was analyzed by fitting the Williamson–Hall equation. Combined with catalytic degradation experiments of methylene blue via the Fenton reaction, the influence of Fe_3_O_4_ grain size on the catalytic performance of the composite was elucidated. Under different preparation conditions, the Fe_3_O_4_ grain size in the composites exhibited distinct variation characteristics. With an increase in the Fe_3_O_4_ loading ratio, the Fe_3_O_4_ grain size gradually increased, accompanied by enhanced catalytic degradation performance. When the preparation temperature was varied, the Fe_3_O_4_ grain size increased with rising temperature, whereas the catalytic degradation performance of the composite gradually declined. Increasing the mechanical stirring speed led to a decrease in the Fe_3_O_4_ grain size, and the catalytic degradation performance of the composite increased accordingly. The results indicate that the Fe_3_O_4_ loading amount, preparation temperature, and mechanical stirring intensity can all regulate the Fe_3_O_4_ grain size in the palygorskite/Fe_3_O_4_ composite. Moreover, loading an appropriate amount of Fe_3_O_4_ particles onto the palygorskite surface and reducing the Fe_3_O_4_ grain size can effectively improve the catalytic degradation performance of the PAL/Fe_3_O_4_ composite.

## 1. Introduction

Grain size is one of the most fundamental structural characteristics of materials and a key factor determining catalytic oxidation performance. Variations in grain size directly affect the catalytic oxidation activity of materials. Gracia et al. [1] demonstrated that, under oxygen-rich conditions, the turnover frequency (TOF) for CO oxidation increases with increasing Pt grain size and that the active surface of the Pt/SiO_2_ catalyst during CO oxidation is metallic Pt. Furthermore, oxidation rates at different sites on Pt grains differ significantly due to their size. Shao et al. [2] directly proved that grain size affects the redox properties of catalysts and the generation efficiency of reactive oxygen species, thereby determining catalytic oxidation activity. Studies have shown that when the grain size of MoO_3_ decreases from 15.8 nm to 5.3 nm, the oxidative desulfurization (ODS) performance of the catalyst is significantly enhanced; however, further reduction to 2.8 nm sharply increases the apparent activation energy (Eₐ), leading to a marked decline in catalytic performance. Shang et al. [3] confirmed that precisely controlling the size of spinel oxide (CoMnO_x_) to the single-nanometer scale optimizes its cation distribution, thereby achieving oxygen reduction reaction performance surpassing Pt/C. With continuous advances in nanomaterial research, grain size has demonstrated multifaceted application potential in regulating surface adsorption, separation, catalytic degradation, and related processes.

Palygorskite, a layered-chain clay mineral, has become an ideal catalyst support due to its unique mesoporous structure and abundant surface-active sites [4]. Fe_3_O_4_ nanoparticles combine excellent catalytic performance with magnetic responsiveness, showing broad application prospects in environmental catalysis. Constructing a composite by loading Fe_3_O_4_ onto the palygorskite surface can synergistically utilize the mesoporous adsorption capacity of palygorskite and the catalytic oxidation activity of Fe_3_O_4_, enabling efficient degradation of organic pollutants [5]. The grain size of nanoparticles has a decisive influence on their physicochemical properties and is a key tuning parameter for the catalytic oxidation performance of composites [6,7,8]. For the PAL/Fe_3_O_4_ composite, the Fe_3_O_4_ grain size largely determines the catalytic performance of the material; however, accurately measuring the grain size of nanoparticles remains a challenge.

In existing studies, scanning electron microscopy (SEM), transmission electron microscopy (TEM), and X-ray diffraction (XRD) analysis can all accurately determine the grain size of micron-sized particles; however, their measurement accuracy for nanoparticles is far from sufficient. Moreover, both SEM and TEM rely on direct imaging observations, and the obtained nanoscale dimensions represent only local grain sizes rather than the overall grain size distribution of the sample. As an important tool for material characterization, X-ray diffraction analysis provides rich microstructural information from its diffraction line profiles. Since Max von Laue discovered the X-ray diffraction phenomenon in crystals in 1912, X-ray diffraction has gradually become an indispensable tool for material structural characterization [9]. By measuring the diffraction patterns of X-rays in materials, X-ray diffraction enables the non-destructive acquisition of crystal structure characteristics, including phase composition, crystallite size, and microstrain [10]. In 1953, Williamson and Hall first proposed the concept of decomposing the integral breadth of diffraction peaks into contributions from size and strain [11], which provided a new method for measuring grain size in materials exhibiting asymmetric X-ray diffraction peaks [12]. Compared to the Scherrer equation, which only uses integral breadth while neglecting the contribution of strain, this method offers a more accurate calculation of grain size for minerals with asymmetric peak profiles.

In summary, considering the asymmetric X-ray diffraction peak profiles of the PAL/Fe_3_O_4_ composite, this study employs the Williamson–Hall (W.-H.) method. Using the PAL/Fe_3_O_4_ composite prepared by co-precipitation, combined with the Rietveld whole-pattern fitting module of the Highscore 5.7 software, as well as the Cagliotti equation and the Pearson IV equation, we conducted quantitative analysis of the Fe_3_O_4_ grain size in the PAL/Fe_3_O_4_ composite and elucidated the effect of grain size on the catalytic performance of the material.

## 2. Materials and Methods

### 2.1. Purification of Palygorskite

**Figure 1:**
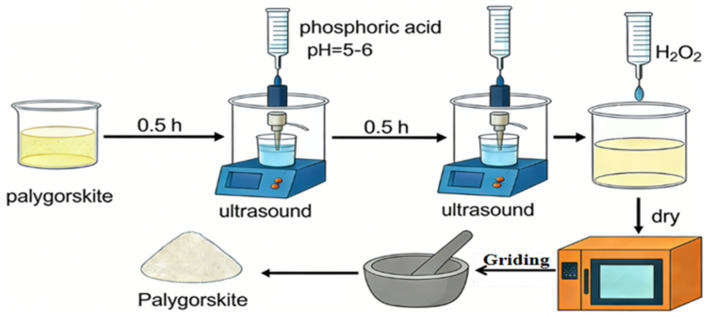
Schematic diagram of the palygorskite purification process.

The raw palygorskite ore was collected from Dafang County, Bijie City, Guizhou Province, China. The purification of palygorskite was carried out using a combined acid leaching and ultrasonication method. The purification process is illustrated in Figure 1. The palygorskite was placed in a round-bottom flask, and distilled water was added at a mass ratio of 10:1 (water-to-palygorskite ratio). After ultrasonic dispersion for 30 min, a 1 mol/L H_3_PO_4_ solution was added until the pH reached 4–5. Following thorough ultrasonic dispersion, excess hydrogen peroxide (H_2_O_2_) was added until no further bubbles were generated in the solution system. The mixture was then centrifuged, and the resulting product was dried at 70 °C.

### 2.2. Preparation of Pal/Fe_3_O_4_ Composites

**Figure 2:**
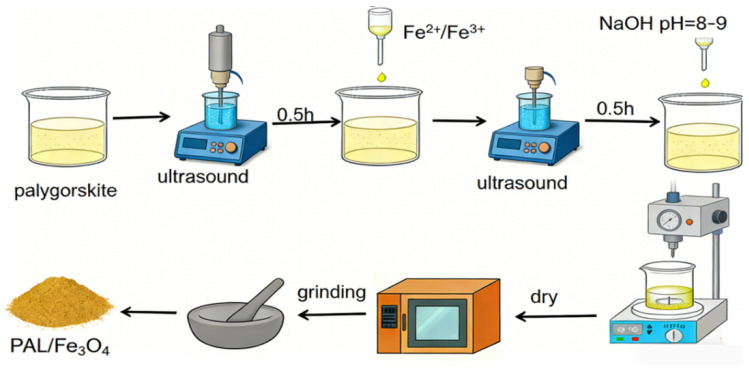
Preparation diagram of PAL/Fe_3_O_4_ composite.

The preparation process of the PAL/Fe_3_O_4_ composites is illustrated in Figure 2. The purified palygorskite was added to deionized water at a solid-to-liquid ratio of 1:10 and ultrasonically dispersed for 45 min. Subsequently, a 0.2 mol/L FeCl_3_ solution was added dropwise under ultrasonic mixing for 15 min to facilitate the adsorption of Fe^3+^ onto the acidic sites of palygorskite. Then, a 0.45 mol/L FeCl_2_ solution was introduced, followed by ultrasonic stirring for 45 min. A 0.5 mol/L NaOH solution was added dropwise until the pH reached 8–9, and the mixture was subjected to continuous ultrasonication for 60 min to promote the conversion of the colloid into Fe_3_O_4_ nanoparticles. The resulting product was centrifuged at 1500 r/min for 15 min, washed three times with anhydrous ethanol, and dried at 40 °C for 9 h to obtain the target composite.

The preparation conditions were as follows: ① To vary the PAL-to-Fe_3_O_4_ mass ratio, the temperature was kept at 25 °C and the mechanical stirring speed at 250 r/min. Composites with PAL:Fe_3_O_4_ ratios of 4:1, 3:1, 2:1, and 1:1 were prepared and designated as L-4, L-3, L-2, and L-1, respectively. ② To vary the synthesis temperature, the stirring speed was kept at 250 r/min and the PAL:Fe_3_O_4_ mass ratio at 3:1. Composites prepared at 25 °C, 40 °C, 55 °C, and 70 °C were designated as T-25 °C, T-40 °C, T-55 °C, and T-70 °C, respectively. ③ To vary the mechanical stirring speed during synthesis, the temperature was kept at 25 °C and the PAL:Fe_3_O_4_ mass ratio at 3:1. Composites prepared at stirring speeds of 250, 500, 750, and 1000 r/min were designated as S-250, S-500, S-750, and S-1000, respectively.

### 2.3. Characterization Methods of the Composites

#### 2.3.1. XRD Phase Analysis and Peak Profile Study

X-ray diffraction (XRD) patterns were collected using a Panalytical X’Pert Pro diffractometer (Cu *Kα* radiation, *λ* = 0.15406 nm), which was manufactured by Panalytical Company, Kassel, Germany. Background subtraction, *Kα*_2_ stripping, and phase matching were performed using HighScore Plus 5.1 software against the ICDD PDF-2023 database to obtain semi-quantitative phase compositions and lattice parameters.

#### 2.3.2. Grain Size Analysis

Based on the variation in the full width at half maximum (FWHM) of XRD peaks, the grain size was calculated using the Scherrer equation (Equation (1)) [13]:

(1) $$d_{hkl}=\frac{k\lambda }{\beta \cos\theta }$$

where $d_{hkl}$ is the grain size at the (hkl) crystal plane of the sample; *λ* is the X-ray wavelength (Cu *Kα* radiation, λ = 0.154 nm); *β* is the FWHM of the diffraction peak; *θ* is the Bragg angle; and k is the Scherrer constant (dimensionless, with its value varying depending on the grain shape of the material: for spherical grains, k = 0.891; for tetrahedral grains, k = 0.73~1.03; for octahedral grains, k = 0.82~0.94). It should be noted that the Scherrer equation is applicable only to symmetric peak profiles [14].

Taking the logarithm of both sides of Equation (1) yields the Williamson–Hall (W.-H.) equation, as shown in Equation (2):

(2) $$\ln\left(\beta \right)\cos\theta =\frac{k\lambda }{D}+ln(\frac{1}{\cos\theta })$$

By incorporating the internal stress of the material, Equation (2) can be transformed into the linear W.-H. equation [15,16]:

(3) $$\beta \cos\theta =\frac{k\lambda }{D}+4\varepsilon \sin\theta$$

where ε is the microstrain and *D* is the grain size.

In the fitting procedure of this experiment, Equation (3) was adopted using HighScore 5.7 software, combined with the Pearson IV function and FWHM fitting, and employing the Caglioti equation, thereby achieving an accurate calculation of the magnetite grain size in the composite.

#### 2.3.3. Catalytic Performance Testing of Pal/Fe_3_O_4_ Composites and Mb Removal

Based on the mechanism of the Fenton reaction, the catalytic performance of the PAL/Fe_3_O_4_ composite material was evaluated by combining spectrophotometric analysis. The detailed procedure was as follows: 0.07 g of PAL/Fe_3_O_4_ materials prepared under different conditions were accurately weighed and placed into 10 mL centrifuge tubes, respectively. Subsequently, 5 mL of 200 mg/L methylene blue (MB) solution and 3 mL of 1 mol/L HCl solution were added to adjust the pH value to 2–3. Afterwards, a 1 mol/L H_2_O_2_ solution was added to initiate the Fenton reaction. After a certain reaction time, the solution was centrifuged at 1500 r/min, and the supernatant was collected. The residual concentration of MB in the supernatant was determined at a wavelength of 664 nm.

The Fenton process is based on the catalytic decomposition of hydrogen peroxide (H_2_O_2_) by ferrous ions (Fe^2+^), which generates highly reactive hydroxyl radicals (•OH) that can non-selectively oxidize and degrade organic pollutants. The core reaction equations are presented as follows [17]:

Fe^2+^ + H_2_O_2_ → Fe^3+^ + OH + •OH

Fe^3+^ + H_2_O_2_ → Fe^2+^ + H^+^ + •OOH

As shown in the above equations, Fe^2+^ is regenerated in the second reaction, thereby maintaining the continuous operation of the catalytic cycle. The hydroxyl radical (•OH) produced in the reaction has a high oxidation potential (2.70 V), which is the main active substance responsible for the degradation of organic pollutants.

According to the aforementioned literature, the removal efficiency (*η*, %) of pollutants (e.g., dyes and chemical oxygen demand (COD)) in the Fenton process is calculated using the following formula [18]:

$$\eta =\frac{C_{0}-C_{t}}{C_{0}}$$

where *η* represents the pollutant removal efficiency (%), *C*_0_ is the initial concentration of the pollutant (mg/L or mol/L), and *C_t_* is the residual concentration of the pollutant at reaction time *t* (mg/L or mol/L).

## 3. Results and Discussion

### 3.1. Phase Composition of Purified Pal and Pal/Fe_3_O_4_ Composites

**Figure 3:**
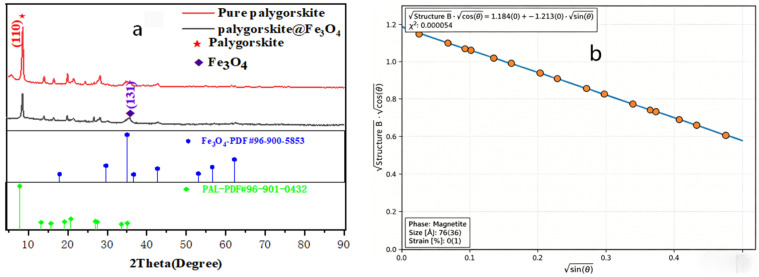
X-ray diffraction pattern of the material. (**a**) The material composition of PAL and Pal/Fe_3_O_4_ composite; (**b**) the fitting diagram of W-H dimension.

The phase compositions of the purified PAL and sample L-3 (PAL:Fe_3_O_4_ = 3:1) composite are shown in Figure 3a. As can be seen, PAL exhibited characteristic diffraction peaks at 8.53°, 14.00°, 16.47°, 18.30°, 21.44°, 27.54°, 28.06°, and 35.54°, which are consistent with the standard peaks of palygorskite from the International Crystal Diffraction Database (ICDD PDF 96-901-0432). The characteristic peaks of the PAL/Fe_3_O_4_ composites remained largely consistent with those of PAL. Weak peaks corresponding to Fe_3_O_4_ crystals (PDF 96-900-5838) appeared at 28.30° and 30.16°. Moreover, the peak at 35.54°, assigned to the (131) plane of Fe_3_O_4_, became sharper, indicating a high degree of crystallinity and confirming that Fe_3_O_4_ nanoparticles were successfully loaded onto the palygorskite surface [14].

According to the W–H fitting results (Figure 3b), the XRD peaks of the magnetite (Fe_3_O_4_) samples exhibited pronounced asymmetric characteristics [19,20]. The average grain size of the samples was approximately 7.6 nm (76 Å), which falls within the typical nanocrystalline regime. This asymmetry is attributed to the non-uniform size distribution of nanoscale grains. Furthermore, lattice microstrain and crystal defects also serve as evidence for the peak profile distortion of magnetite [21].

### 3.2. Effect of Different Preparation Conditions on the Grain Size of Fe_3_O_4_

#### 3.2.1. Effect of Loading Ratio on the Fe_3_O_4_ Grain Size in the Composites

**Figure 4:**
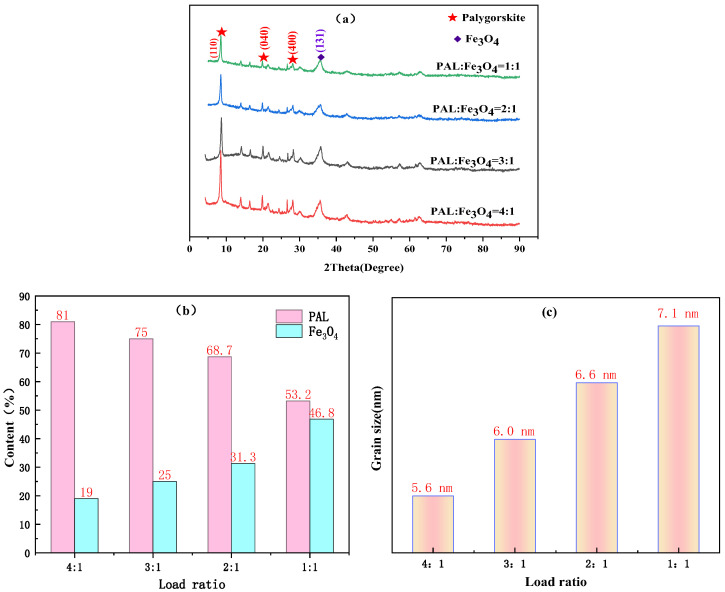
Analysis and comparison diagram of different load ratios. (**a**) XRD diagram of PAL and Fe_3_O_4_ load ratio; (**b**) the phase composition distribution; (**c**) Fe_3_O_4_ grain size diagram.

In composite materials, the content of the reinforcing phase (i.e., the loading ratio) is a critical factor affecting the grain size of the metal matrix, and the underlying mechanism is attributed to its anchoring effect on grain boundaries. In this study, the Rietveld refinement module embedded in HighScore Plus 5.1 software was adopted to fit the phase composition based on the XRD patterns presented in Figure 4a. and the corresponding fitting results are displayed in Figure 4b.

The results demonstrate that as the proportion of PAL in the loading ratio increased, the relative content of magnetite decreased correspondingly. Specifically, at a PAL:Fe_3_O_4_ mass ratio of 1:1, the contents of palygorskite and magnetite were 81.0% and 19.0%, respectively; at a ratio of 2:1, the contents were 75.0% and 25.0%, respectively; at a ratio of 1:3, the contents were 68.7% and 31.3%, respectively; and at a ratio of 4:1, the contents were 53.2% and 46.8%, respectively. Grain size analysis of the Fe_3_O_4_ phase in the PAL/Fe_3_O_4_ composites was conducted using a grain size fitting method. This method was based on Equation (3) combined with the Caglioti equation to establish an analytical full width at half maximum (FWHM) model, and the results are shown in Figure 4c.

With the continuous increase in the magnetite proportion, the grain size of magnetite loaded on the palygorskite surface exhibited a slowly increasing trend. When the PAL:Fe_3_O_4_ ratio was 4:1, the Fe_3_O_4_ grain size on the palygorskite surface was 5.6 nm. As the loading ratio further increased, the grain size gradually increased; when the PAL:Fe_3_O_4_ ratio increased to 1:1, the Fe_3_O_4_ grain size reached 7.1 nm, corresponding to an overall increase of 1.5 nm.

#### 3.2.2. Effect of Preparation Temperature on Fe_3_O_4_ Crystal Size

**Figure 5:**
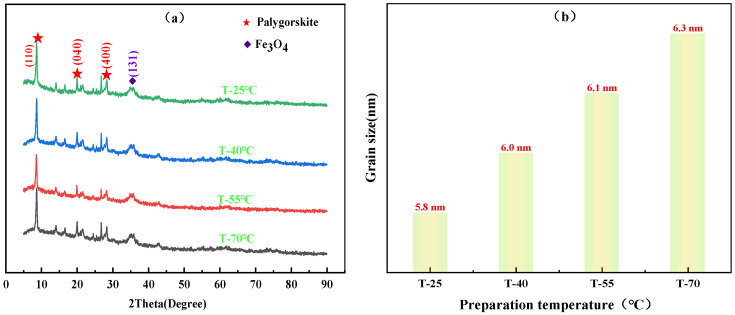
Fitting plots of magnetite grain size at different preparation temperatures. (**a**) XRD patterns of the composites at different temperatures; (**b**) the variation in magnetite grain size with temperature.

Figure 5 shows the XRD patterns of the PAL/Fe_3_O_4_ composites prepared at different temperatures and the corresponding Fe_3_O_4_ grain sizes. The fitting results indicate that as the preparation temperature increased, the magnetite grain size in the composites gradually increased. The smallest grain size, 5.8 nm, was obtained in the composite prepared at 25 °C, while the largest grain size, 6.3 nm, was achieved at 70 °C. This suggests that a higher temperature facilitates grain growth on the support surface, thereby increasing the grain size. This phenomenon may be attributed to the requirement of thermal kinetic energy during the growth of magnetite particles on the palygorskite surface to promote grain migration at the palygorskite interface. Increasing the temperature can enhance atomic diffusion to a certain extent, thus promoting grain growth [22].

#### 3.2.3. Effect of Mechanical Stirring on the Grain Size of Magnetite

In materials science, the sharpness of X-ray diffraction (XRD) peaks is defined as peak broadening, which is mainly governed by grain purity and lattice periodicity, namely, large grain size and low microstrain. Stirring is a high-energy mechanical treatment that induces numerous lattice defects, including dislocations and vacancies, and further refines grain particles. When the stirring speed increases from 250 r/min to 500–1000 r/min, the input mechanical energy rises remarkably. Severe mechanical impact effectively refines grains and accumulates abundant microstrain.

In accordance with the Scherrer equation, a smaller grain size leads to more obvious XRD peak broadening, and increased microstrain also promotes such broadening. Although low-speed stirring at 250 r/min can realize grain refinement, the resultant grains possess a larger particle size and superior lattice integrity relative to those synthesized under high stirring rates. Accordingly, the characteristic peaks present a smaller full width at half maximum and thus sharper profiles in XRD spectra.

**Figure 6:**
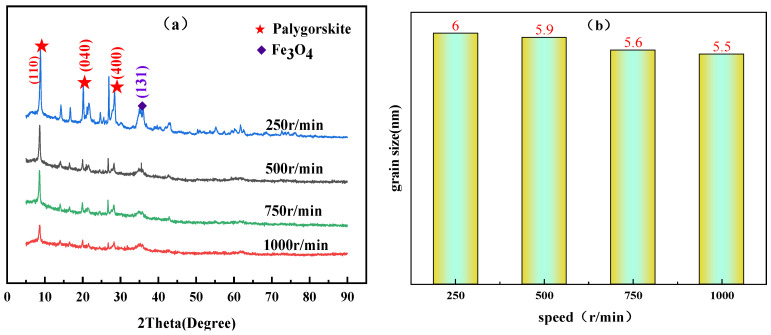
Analysis diagram of Pal/Fe_3_O_4_ composite prepared by mechanical stirring. (**a**) XRD diagram of the composite material; (**b**) the change diagram of Fe_3_O_4_ grain size.

Figure 6a,b display the XRD patterns and grain size variation in PAL/Fe_3_O_4_ composites prepared at different stirring speeds. The distinct diffraction peak assigned to the (131) crystal plane of magnetite appeared at 2θ = 35.54°. Williamson–Hall fitting results revealed that the grain size of magnetite in composites gradually declined with increasing stirring speed during synthesis. The maximum grain size of 6.0 nm was obtained at 250 r/min, while the minimum value of 5.5 nm was achieved at both 750 r/min and 1000 r/min. This phenomenon is ascribed to the fact that mechanical stirring enables the uniform dispersion of Fe^2+^/Fe^3+^ ions.

### 3.3. Effect of Fe_3_O_4_ Grain Size on the Catalytic Performance of the Composites

**Figure 7:**
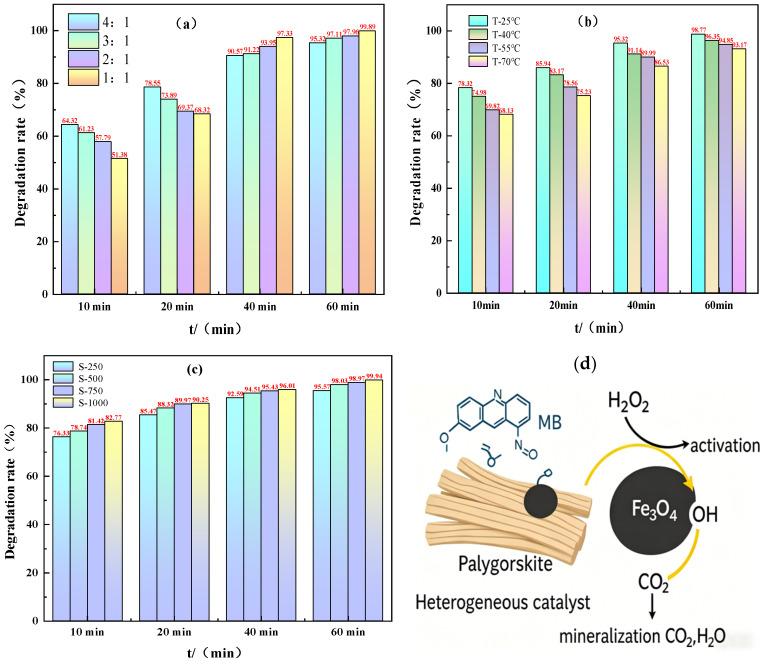
Degradation of MB by Pal/Fe_3_O_4_ composite under different preparation conditions. (**a**) Different load ratios; (**b**) different preparation temperatures; (**c**) different stirring speeds, and (**d**) mechanism diagram of MB removal by composite material.

The effects of magnetite grain size, tuned by varying the loading ratio, synthesis temperature, and mechanical stirring speed, on the catalytic degradation performance of PAL/Fe_3_O_4_ composites were investigated. Figure 7a shows the degradation profiles of methylene blue (MB) by the composites prepared under different loading ratios. As illustrated, a higher proportion of magnetite led to better catalytic degradation performance of the composite toward MB. In contrast, for composites prepared at different temperatures (Figure 7b), the catalytic degradation efficiency gradually decreased with increasing preparation temperature. According to the grain size analysis of the composites in Section 3.1, the grain size of magnetite loaded on the palygorskite surface increased with increasing temperature. Thus, a smaller grain size corresponds to stronger catalytic performance of the composite. This conclusion is further supported by the MB degradation results of composites prepared under different stirring conditions (Figure 7c): as the stirring speed increased, the magnetite grain size decreased, and the degradation efficiency of MB improved. This behavior is related to the Fenton reaction process. As illustrated in Figure 7d, during the reaction, small Fe_3_O_4_ grains on the PAL surface accelerated interfacial electron transfer due to their high specific surface area, promoting the generation of hydroxyl radicals [23,24], and the reaction rate continuously increased. In contrast, large magnetite grains suffered from insufficient catalytic activity, and the reaction rate gradually decreased. In summary, a higher loading amount of magnetite and a smaller grain size both lead to stronger catalytic activity of the PAL/Fe_3_O_4_ composite [25].

## 4. Conclusions

In this study, the Williamson–Hall analysis method was successfully applied to quantify the grain size of magnetite in PAL/Fe_3_O_4_ composites. The phase composition and magnetite grain size of the composites prepared under different loading ratios, synthesis temperatures, and mechanical stirring speeds were quantitatively analyzed, and the effect of grain size on the catalytic activity of the composites was investigated. The XRD analysis confirms the successful loading of Fe_3_O_4_ onto the palygorskite surface. The grain size of Fe_3_O_4_ can be effectively tuned by adjusting the synthesis conditions: increasing the Fe_3_O_4_ loading ratio or raising the preparation temperature leads to an increase in grain size (from 5.6 nm to 7.1 nm and from 5.8 nm to 6.3 nm, respectively). In contrast, increasing the mechanical stirring speed effectively reduces the magnetite grain size from 6.0 nm to 5.5 nm. Catalytic performance tests demonstrate that the Fe_3_O_4_ grain size is a key factor influencing the catalytic degradation performance of the composite toward methylene blue. The catalytic activity of PAL/Fe_3_O_4_ can be improved by a smaller grain size. In summary, the catalytic activity of the PAL/Fe_3_O_4_ composite can be effectively enhanced by reducing the Fe_3_O_4_ grain size through the use of a higher mechanical stirring speed and a moderately low preparation temperature.

## Data Availability

The original contributions presented in this study are included in the article. Further inquiries can be directed to the corresponding author.
